# Deep Residual Networks for User Authentication via Hand-Object Manipulations

**DOI:** 10.3390/s21092981

**Published:** 2021-04-23

**Authors:** Kanghae Choi, Hokyoung Ryu, Jieun Kim

**Affiliations:** ImagineX Lab, Graduate School of Technology and Innovation Management, Hanyang University, Seoul 04763, Korea; kanghaechoi@hanyang.ac.kr (K.C.); hryu@hanyang.ac.kr (H.R.)

**Keywords:** user authentication, user behaviour, hand movement, IMU-based wearable device, convolutional neural network, behavioural biometrics

## Abstract

With the ubiquity of wearable devices, various behavioural biometrics have been exploited for continuous user authentication during daily activities. However, biometric authentication using complex hand behaviours have not been sufficiently investigated. This paper presents an implicit and continuous user authentication model based on hand-object manipulation behaviour, using a finger-and hand-mounted inertial measurement unit (IMU)-based system and state-of-the-art deep learning models. We employed three convolutional neural network (CNN)-based deep residual networks (ResNets) with multiple depths (i.e., 50, 101, and 152 layers) and two recurrent neural network (RNN)-based long short-term memory (LSTMs): simple and bidirectional. To increase ecological validity, data collection of hand-object manipulation behaviours was based on three different age groups and simple and complex daily object manipulation scenarios. As a result, both the ResNets and LSTMs models acceptably identified users’ hand behaviour patterns, with the best average accuracy of 96.31% and F1-score of 88.08%. Specifically, in the simple hand behaviour authentication scenarios, more layers in residual networks tended to show better performance without showing conventional degradation problems (the ResNet-152 > ResNet-101 > ResNet-50). In a complex hand behaviour scenario, the ResNet models outperformed user authentication compared to the LSTMs. The 152-layered ResNet and bidirectional LSTM showed an average false rejection rate of 8.34% and 16.67% and an equal error rate of 1.62% and 9.95%, respectively.

## 1. Introduction

### 1.1. Backgrounds

The increasing adoption of biometrics in mobile devices has reduced the prevalence of traditional knowledge-based authentication methods, such as personal identification numbers (PINs), passwords, and pattern locks [1]. Biometric authentication can be divided into two types: explicit and implicit [2]. Explicit biometric authentication uses physiological characteristics (e.g., fingerprints, irises, and face shapes) to verify the claimed identity of a user. Despite its simplicity and popularity, such explicit biological information collection raises concerns about privacy and may even lead to the leakage of personal information [3]. Moreover, similar to knowledge-based authentication, explicit biometrics only authenticate a user at the initiation of a device or service, posing significant vulnerability to security attacks that might occur after the initial entry-point authentication [4,5]. In juxtaposition, implicit authentication, which authenticates users based on behaviour patterns, enables implicit, continuous authentication as a background function in a device or service.

As mobile devices become more technologically advanced, built-in sensors, such as accelerometers, gyroscopes, magnetometers, and global positioning system(GPS) [4,6,7], facilitate the collection of users’ behavioural data. A handful of research efforts on implicit behaviour biometrics have been made to enhance user authentication on mobile devices. Suggested solutions include the use of touch gestures [4], keystroke dynamics [8,9], gait patterns [9], and users’ mobile usage behaviours (such as authentication based on how a user connects to a Wi-Fi network or applications at different locations and times) [5,7]. Such methods can complement the current explicit authentication method to authenticate legitimate users after the initial login. These methods exhibit attributes of greater usability and learnability for authenticating users, given that implicit authentication does not require a specific process [1,3].

While the task of user authentication using sensors built into smartphones has been well addressed in the literature, studies on wearable motion sensor-based biometrics are very few [10,11]. The use of wearable motion sensors for user authentication is particularly valuable: first, the incorporated wearable devices for user authentication can come in different forms, such as smartwatches, gloves, or shoe insoles [10,11], which helps to reduce the dependence of user authentication on smartphone devices; secondly, it is important to detect an interaction between fingers and hands on hand-object manipulations [12], but built-in smartphone sensors cannot detect finger movements; thirdly, it enables silent user authentication through the daily use of objects the way the user manipulates objects “in-the-wild” [13] (e.g., lifting a cup or spreading butter with a knife).

### 1.2. Research Aims

The present study aims to investigate state-of-the-art deep residual learning models (ResNets) with multiple depths (i.e., 50, 101, and 152 layers) to explore the potential use of user authentication via hand-object manipulations. Data collection relies on inertial measurement unit (IMU)-based wearable sensors to capture users’ fine-motor finger and wrist movement sequences. Hand behaviour patterns in object manipulation may vary according to the subject’s age and task complexity. Two experiments were performed: (1) We first trained an age-group estimator for users in their 20 s, 50 s, and 80 s on a simple and repetitive object manipulation scenario, using a targeted box and blocks test (tBBT). The tBBT is clinically devised to investigate gross and fine motor hand functions in which a user is required to perform controlled object manipulation scenarios, consisting of grasping, transporting, and releasing small blocks [14]; (2) The second experiment was designed to increase the study’s ecological validity, with more complex and natural sequential hand movements, i.e., smartphone manipulation. Based on the trained dataset, we evaluated the performance of ResNets (ResNet-50, ResNet-101, and ResNet 152) and two long short-term memory (LSTM) models (simple LSTM and bidirectional LSTM) with respect to age group classification and user authentication.

### 1.3. Structure of the Paper

The remainder of this article is structured as follows: Section 2. presents literature reviews on state-of-the-art deep learning approaches in hand behaviour authentication. Section 3. describes the experimental setting explaining the data collection and pre-processing flow to transform the raw sensory data collected through a wearable IMU system. In Section 4., the detailed experimental results are presented. Section 5. discusses the authentication performances and implications of the different deep-learning approaches employed in the study. Finally, Section 6. concludes by addressing research limitations and suggesting directions for future work.

## 2. Related Work

Identifying hand behaviour patterns in object manipulation is challenging. This is mainly due to the wide variety of feature extraction and expensive computational calculations on weight adjustment to build an accurate prediction model [13,15]. Deep learning-based methods have emerged to extract these complex hand behaviour features through raw data from wearable motion sensors (e.g., accelerometers and gyroscope sensors) [16]. Compared to classic machine learning algorithms (e.g., support vector machine (SVM), k-nearest neighbour algorithm (k-NN) [17]), deep learning models use neural networks with multiple layers of non-linear operations to learn data features [18]. The ability of deep learning to automatically learn the most salient data representation without manual feature engineering makes it suitable for sensor data [19].

LSTMs [4], specific types of recurrent neural networks (RNNs), have been widely deployed in the field of gait biometrics [11,20]. An LSTM-based deep network architecture efficiently captures long-term temporal features from multivariate time-series data derived from motion sensors [17]. Recently, Abuhamad et al. [4] proposed LSTM-based hand behavioural pattern authentication models with an embedded sensor in smartphones, and demonstrated that the RNN-based deep learning-based model is applicable for hand behaviour authentication by achieving both false acceptance rates (FAR) and equal error rates (EER) less than 1% with 1 s of hand movement data.

However, given that hand behavioural patterns are characterised in both short-term patterns (per sampling rate) and long-term patterns (per sequence length), there is a trade-off between sequence lengths and authentication times. The LSTM-based hand behavioural pattern authentication models can be very conditional to understand complex hand behavioural patterns, given that the LSTMs have to satisfy the Markov property to conclude a result [21]. That means that the Markov property, in which a result in time *t*_*n*_ is derived only from a result in time *t*_*n*−1_, requires LSTMs to memorise every short-term (i.e., *t*_*n*−1_, *t*_*n*−2_, …, *t*_1_, *t*_0_) data value to comprehend the long-term (*t*_*n*_) pattern data. Consequently, because increasing the sampling rates of sensors may lengthen the hand behavioural pattern sequence, the longer hand movement sequences may need a significantly longer time to authenticate users.

Convolutional neural networks (CNNs), on the other hand, efficiently serve short-term data along with long-term data. A CNN architecture proposed by Kim [22] enables classifying sequential data, such as natural languages, time-series signals, with convolution filters (also known as short-term dependent filters). The convolution filter scans sequential data as an n-gram filter in natural language processing (NLP), which searches a short word sequence within a sentence and slices it until the filter reaches the end of sentences to comprehend the context of the sentence.

Similarly, in behaviour pattern identifications, convolution filters work as short-term dependent filters in CNN with no need to record all short-term data. Mekruksavanich and Jitpattanakul [23] have demonstrated that CNNs outperform user identifications compared to the LSTM-based approaches. Based on the human activity recognition dataset from the University of California, Irvine (UCI HAR), six daily activities were divided into two activities: (1) dynamic activity and (2) static activity. The accuracies of CNN and LSTM were 66.55% and 18.87%, respectively. The result with the dynamic activity dataset demonstrated that CNN can be more efficient than LSTM to comprehend human activities as sequential data.

More recently, a deep residual network (ResNet)—a state-of-the-art CNN model—has received attention because it can handle short-term sequences better than traditional CNN models while increasing network depth [24]. ResNet is a residual CNN model proposed by Microsoft researchers in 2015 [24] which can contain considerably deeper convolutional layers with ‘residual connections’ while alleviating the problem of vanishing gradient on traditional CNN architecture [24,25]. In addition, ResNet features easier optimisation, computational efficiency, and high accuracy gains with increased network depth [24]. For instance, in multi-touch sequential gesture recognition, Debard et al. [21] compared the performance of LSTMs and CNNs. The results empirically confirmed the improved recognition rate of the CNN models (89.96% accuracy in CNN [21] vs. 73.10% in simple LSTM [4] and 87.72% in 2D-LSTM [26].

In this regard, this paper introduces the state-of-the-art CNN-based ResNet models with a depth of up to 152 layers for user authentication in hand-object manipulation and compared its performance against the benchmark RNN-based LSTMs (simple LSTM and bidirectional LSTM).

## 3. Methods

### 3.1. Wearable IMU System for Collecting User’s Hand Behaviour Data during Object Manipulation

**Figure 1:**
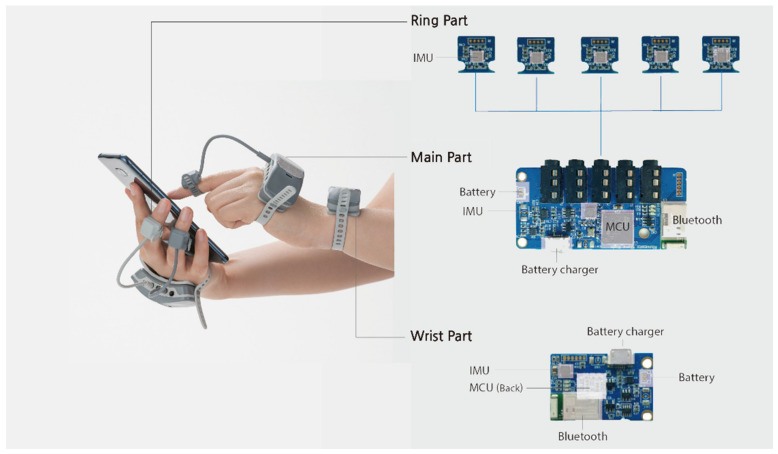
Wearable inertial measurement unit(IMU) system for collecting hand movement data during object manipulation.

A clip-on wearable IMU-based system [27] was used in this study to capture users’ fine-motor finger and wrist joint movements while manipulating objects. The system comprises IMU-embedded hand and wrist modules that can be selectively applied to the fingers and wrist (see Figure 1). Its form factor is particularly suitable for the present study, in the sense that inter-finger interferences can be significantly reduced by eliminating undesirable contact on bare hands, thereby increasing the precision of hand-object manipulation. Each module contained a 9-axis IMU sensor (a 3-axis accelerometer, 3-axis gyroscope, and 3-axis magnetometer) to quantify the dynamic movements of the finger and wrist joints into discrete signals.

Algorithms related to the IMU-based wearable system such as correcting misalignment and joint estimation error are based on our previous paper [27]. To reduce possible misalignment between the IMU sensor and the earth frame, known as the drift phenomenon, the IMU-based wearable system embedded a gradient descent algorithm proposed by Madgwick et al. [27,28]. To estimate realistic joint angle between a pair of IMU sensors, the equation proposed by Brennan et al. [27,29] was used to correct joint estimation errors due to mounting the IMU sensors upon a subject’s hand.

(1) $$Flexion∕Extension\left(\alpha \right)=\tan^{-1}\left(\frac{\cos\beta \times \sin\alpha }{\cos\beta \times \cos\alpha }\right)$$

(2) $$Ulnar∕Radial\;flexion\left(\beta \right)=\sin^{-1}\left(\sin\beta \right)$$

**Figure 2:**
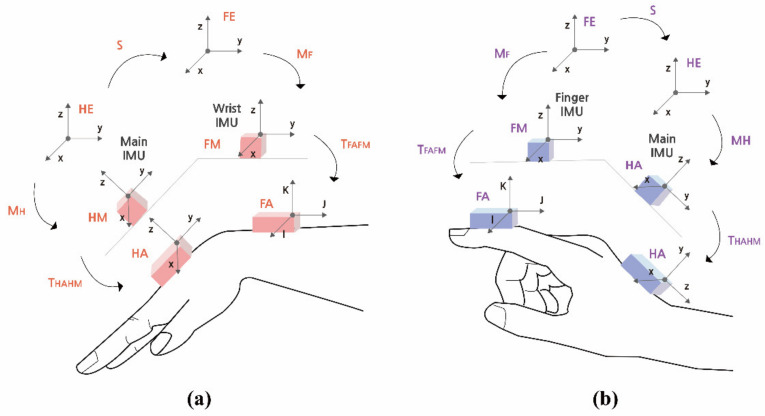
A coordinate system related to joint angle estimation: (**a**) hand to wrist; (**b**) finger to hand [27].

Figure 2a,b shows positions between hand anatomical position (HA) and forearm anatomical position (FA) with sensors attached on both positions. Attached sensors are represented as hand measurements (HM) and forearm measurements (FM), in accordance with the sensor positions. Figure 2 also visualises coordinate systems of both HM and FM based on a subject’s hand position. The sampling rate of the embedded IMU sensor was 100 Hz. Further details of the specifications and mathematical descriptions are provided in our previous paper [27].

### 3.2. Experimental Protocol

Two experiments were designed to collect diverse hand-object manipulation behaviour data and examine the performance of different deep-learning models—ResNets and LSTMs—for user classification and authentication. Experiment 1 aimed to verify age-group classification (20s, 50s, and 80s) and user authentication in the same age group when conducting a simple, controlled object manipulation scenario, i.e., a targeted box and block test. Experiment 2 was designed to increase the ecological validity of Experiment 1 with more complex and natural hand behaviour data, such as grasping, flipping, and releasing a smartphone.

In Experiment 1, 44 subjects in three age groups, i.e., subjects in their 20s (*n* = 18, age = 26.2 ± 3.0), 50s (*n* = 10, age = 54.0 ± 3.0), and 80s (*n* = 16, age = 82.6 ± 3.4), repeated the tBBT three times. We obtained 2032 data values, comprising 766 data points for users in their 20s (38.0%), 527 for users in their 50s (26.0%), and 739 for users in their 80s (36.0%). Next, for user authentication in the same age group, 922 datasets containing 176 registered user data points (19.0%) and 746 unregistered user data values (81.0%) were extracted from 18 subjects in their 20s. All subjects were healthy and right-handed. Participants were briefed about the purpose of the study and signed informed consent before the procedure. The study was reviewed and approved by the Hanyang University Institutional Review Board (HYI-18-142-2, 18 October 2018).

**Figure 3:**
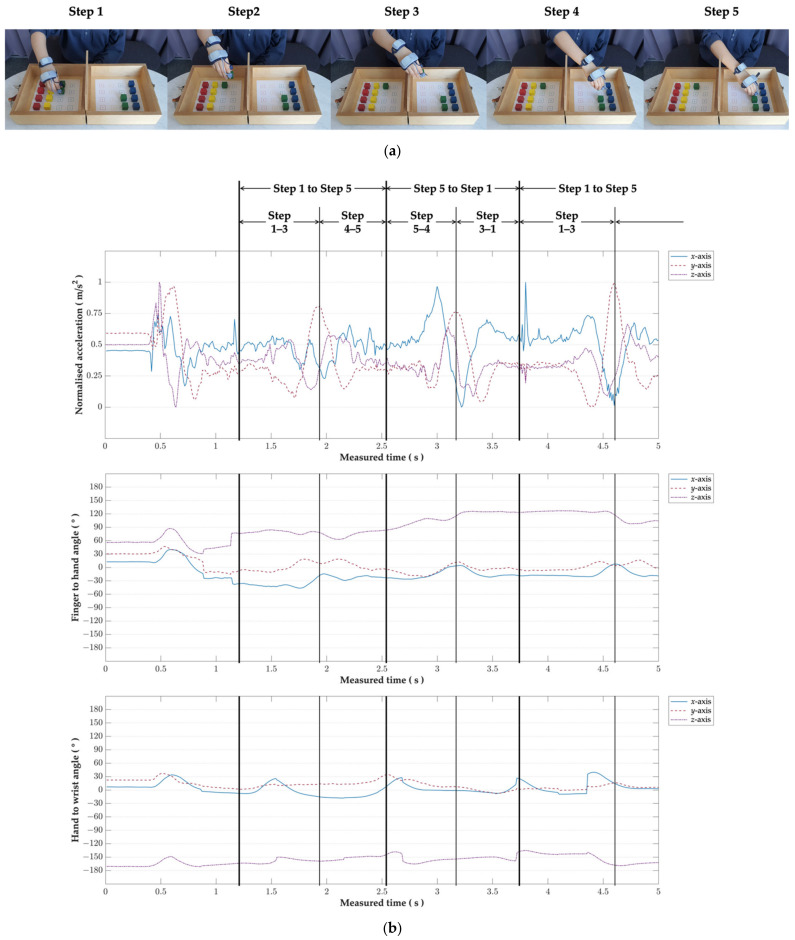
Hand movement sequences in Experiment 1: a targeted box and blocks test. (**a**) Motion sequence depiction in 5 steps; (**b**) IMU sensor output graph and intervals where the five-step motion sequence happens.

The tBBT is a standard hand dexterity test that aims to closely model common real-world object manipulation scenarios, in which a subject is required to control a movement from pick-up to release [27]. As shown in Figure 3a, subjects were asked to transport a total of 16 blocks in a controlled manner, starting from grasping a block in a left box (Step 1–Step 2), transporting it over the partition (Step 2–Step 4), and releasing it in its mirrored position as quickly and accurately as possible (Step 4–Step 5). This simple hand-object manipulation scenario is particularly pertinent for testing the deep learning models in the first place, in that variations in the input dataset are smaller than in the real-world object manipulation scenario, which is a challenge in Experiment 2.

Figure 3b shows an example of the subject’s hand movement with several metrics: the thumb’s normalised acceleration ($Acc_{normalised}$) and the Euler angles for the finger and hand and for the hand and wrist, which corresponds to a series of steps to complete the tBBT. The thumb’s acceleration (Acc) was normalised between 0 to 1 according to Equation (3).

(3) $$Acc_{normalised}=\frac{Acc-Acc_{min}}{Acc_{max}-Acc_{min}}$$

The thumb’s acceleration was regarded as a standard metric to parse the rest of the hand movement data. This is mainly because the hand-object manipulation movement requires a wide range of thumb movement, which engenders a greater extent of changes in the value of the thumb acceleration in comparison to the other hand parts [27]. The following sections explain this in more detail.

While the subject grasped and transported a block over the partition as quickly as possible (i.e., from Step 1 to Step 3), the thumb’s acceleration on each axis tended to increase until the subject completed the steps. On the other hand, during Step 3–Step 5, we observed the trade-off of speed–accuracy in which the subject deaccelerated the hand movement to release the block precisely on the mirrored position. The accelerations in the *x*-, *y*-, and *z*-axis data gradually converged to zero when hand movement reached Step 5.

To repeat the tBBT, repositioning of the subject’s hand position to the initial point is needed. During this process, the acceleration pattern reversed, such as from Step 5 to Step 1. While these backward movement’s data resembled the initial movement, the time spent on hand repositioning became slightly shorter.

The graph with the thumb’s acceleration was then synced with the Euler angler graphs for the finger and hand and for the hand and wrist. The Euler angle for the finger and hand clearly depicts the fluctuation of the Euler angle from transporting a block to the mirrored position to repositioning the hand. Interestingly, the Euler angle for the finger and wrist tended to take a greater value during the repositioning steps (i.e., from Step 5 to Step 1). Considerable variability was observed among the subjects. On the other hand, regarding the Euler angle for the hand and wrist, its extension was smaller than that for the finger and hand but showed a clear up and down.

In Experiment 2, a smartphone manipulation scenario was designed to elicit hand movements that were more similar to those executed in everyday activities. Nine subjects in their 20s (*n* = 9, age = 26.0 ± 3.0) were instructed to perform the scenario “checking the time on the smartphone’s screen” five times. A total of 185 datasets containing 37 registered user data points (20.0%) and 148 unregistered user data points (80.0%) were collected.

**Figure 4:**
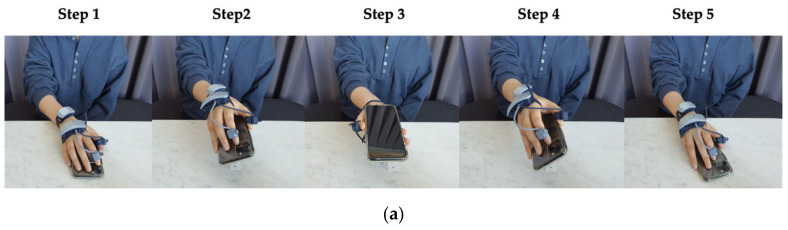

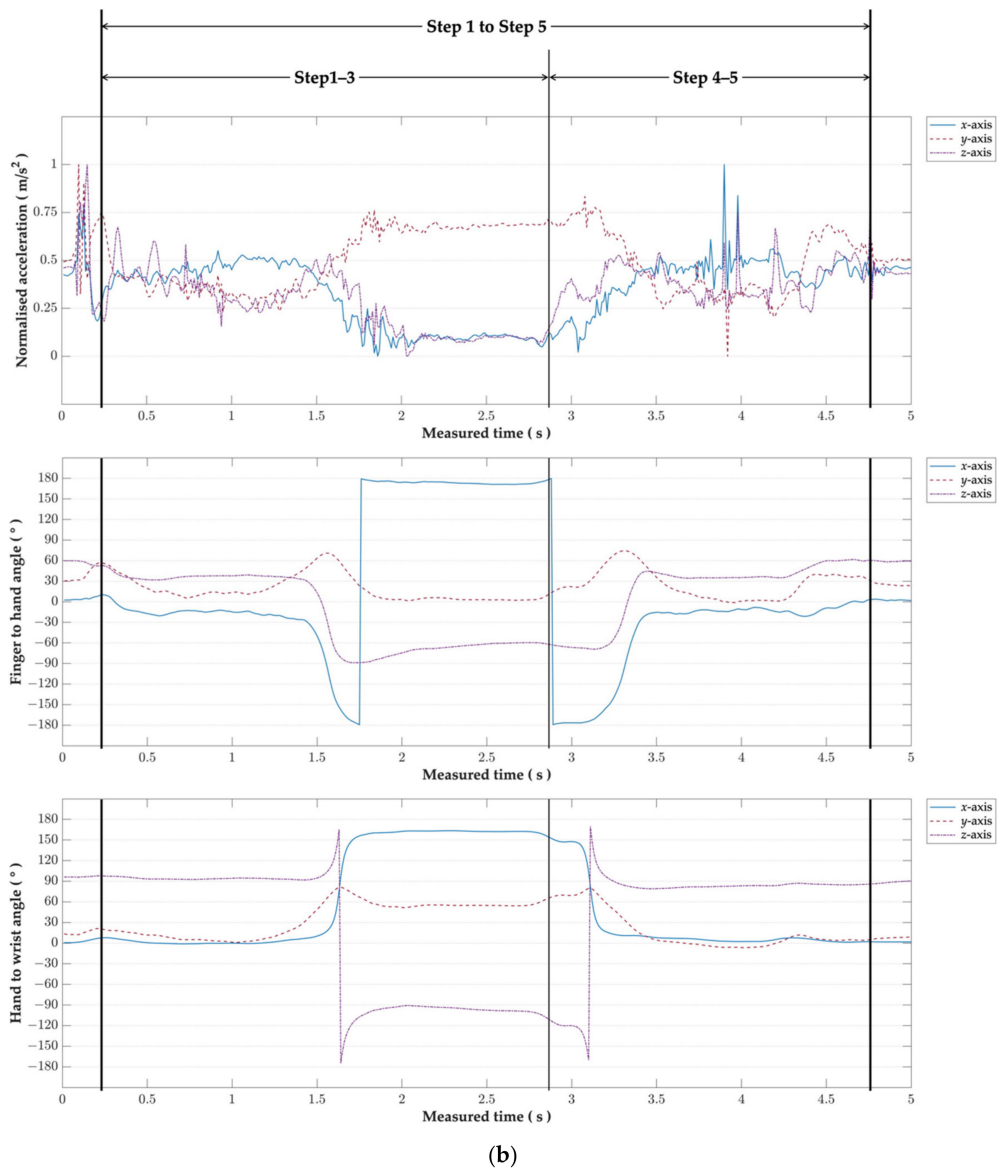
Hand movement sequences in Experiment 2: checking the time on a smartphone’s screen. (**a**) Motion sequence depiction into 5 steps; (**b**) IMU sensor output graph and intervals where the five-step motion sequence happens.

Figure 4a depicts the hand movement sequence for Experiment 2, which consisted of the following five steps: reaching, lifting, flipping, facing down, and placing back a mobile on the table. In juxtaposition to Experiment 1, the subjects were not asked to complete the assigned scenario as quickly as possible. Instead, Experiment 2 aimed to collect subjects’ natural hand-object manipulation movements while increasing the task complexity such as a flipping hand motion (i.e., reversing the smartphone with the screen facing up and down).

As shown in Figure 4b, the subject’s thumb acceleration frequently changed from Step 1 to Step 3. However, we observed a specific period where no change any of the thumb’s axes of acceleration existed: at the transition between Step 3 and Step 4, where the subject held the smartphone to check the time. After checking the time, the second wave in fluctuation followed in Step 4 and Step 5, to put back the smartphone on the table. Other distinguishing features of hand movement in Experiment 2 are in the Euler angle graphs. While Experiment 1 showed gradual change and regularity on the Euler angle graphs, more dynamic changes on both Euler angles (1) between finger and hand, and (2) between hand and wrist were observed in Experiment 2. The Euler angles showed the extension of finger and hand joint to a great degree, in particular, when the subject flipped the smartphone in Step 3.

### 3.3. Flow of User Authentication in Object Manipulation

**Figure 5:**
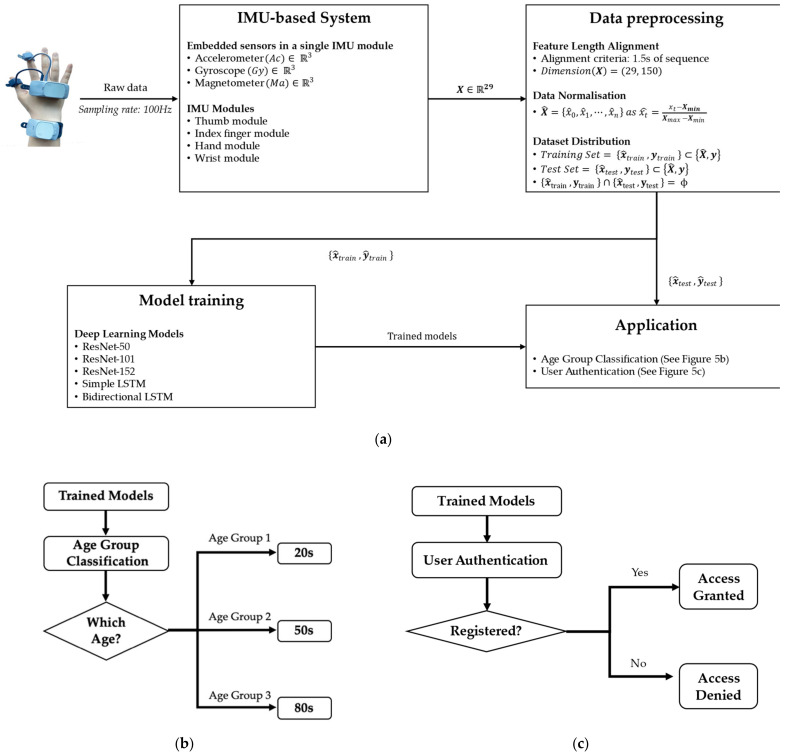
A flow of implementing deep learning-based authentication models: (**a**) A flow of implementing deep learning-based authentication models; (**b**) application: age group classification; (**c**) application: user authentication.

Figure 5a describes the flow of the authentication model, including collecting the wearable IMU sensor data, pre-processing the collected data, and feeding these data to the different deep-learning-based models that are trained to capture the hand behaviour patterns of users in object manipulation, aiming for user classification and authentication. Figure 5b,c describes the system flow of age-group classification and user authentication in practical cases, respectively.

Each subject wore four IMU modules on the thumb, index finger, hand, and wrist on their dominant side. The built-in sensors of the IMU module (9-axis IMU sensor: 3-axis accelerometer, 3-axis gyroscope, and 3-axis magnetometer) captured the subjects’ fine-motor finger and wrist joint behaviour features. During pre-processing, the collected features were aligned to the same sequence length, which contained 150 discrete decimal values, and normalised to reduce any noise produced during data collection. The normalised data were then divided into a training dataset and a test dataset.

In our experiments, we investigated the performance of the age-group classification (Experiment 1) and user authentication (Experiments I and II) using multiple depths of the ResNet model. Three ResNet models were implemented by stacking different numbers of convolution layers: 50, 101, or 152 layers; the ResNet models in this study were named ResNet-50, ResNet-101, and ResNet-152, according to the number of convolution layers contained in the models, respectively. To analyse time-series data used in the present study, we used ResNet’s convolution filter as an n-gram filter to scan hand behaviour features [22]. The n-gram-like convolution filters scanned hand behaviour features at the same time and moved to the features recorded on the next timestamp. By shifting the convolution filters, it constructed convolutional data suitable for CNN architecture. In training ResNet models, the Adam optimiser was utilised with a learning rate of 1 × 10^3^ for the CNN training. Cross-entropy was utilised to predict the labels from the test dataset.

To verify the performance of the ResNet models against the state-of-the-art deep-learning approach, two latest RNN-based LSTM models, named simply LSTM and bidirectional LSTM, were trained and tested. The LSTM model architecture was adopted from the study of Abuhamad et al. [4], in which their high authentication performance was validated based on user behaviour data and smartphone sensor readings.

### 3.4. Performance Metrics

**Figure 6:**
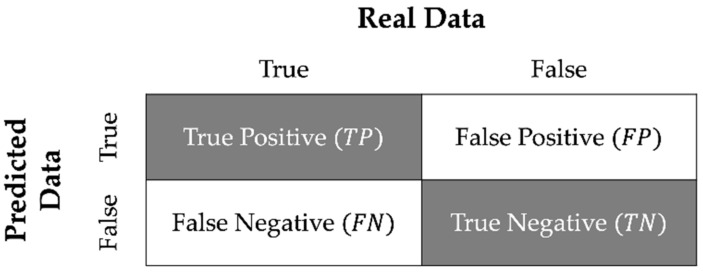
Confusion Matrix.

The performance of deep-learning models was measured based on the indices of the confusion matrix [30]. As Figure 6 shows, there are four indices when comparing real labels and predicted labels in binary classification: true positive (TP), true negative (TN), false positive (FP), and false-negative (FN). Table 1 presents the performance metrics and their equations for authentication models used in the present study, including accuracy, recall, precision, F1 score, false acceptance rate (FAR), false rejection rate (FRR), and equal error rate (EER).

The age-group classification problem in Experiment 1 employs the first four metrics: accuracy, recall, precision, and F1-score. While both accuracy and F1-score have been extensively used in classification tasks, we consider an F1-score as the most pertinent measure in the study because it provides the harmonic means of precision and recall, which emphasises the model’s performance in terms of both false positives and false negatives [4].

Regarding the authentication performance (Experiments I and II), we also calculated the remaining three metrics: FAR, FRR, and EER. FAR is the rate of accepting an imposter biometric sample as a legitimate user. On the other hand, FRR is the rate of incorrectly rejecting a legitimate user as if the user is an imposter. Compared to FRR values, FAR is more dangerous when hacking a system because its authentication model allows illegitimate users to easily access the system. Finally, EER is an approximate value of when FAR and FRR become equal. The present study linearly approximated ERR by calculating the average between FAR and FRR, where the numerical gap between FAR and FRR is minimal.

## 4. Results

### 4.1. Age Group Classification in Experiment 1 (a Targeted Box and Block)

Table 2 summarises the results of the age group classification in Experiment 1. Overall, the deep residual networks (ResNet-50, ResNet-101, and ResNet-152) outperformed the benchmark LSTM models for all metrics. Among the ResNet models, ResNet-50 and ResNet-101 obtained the highest F1-score of 93.04% (93.04% for ResNet-50 and 93.08% for ResNet-101), followed by ResNet-152 (91.84%). Both simple LSTM and bidirectional LSTM models showed F1 scores over 90%, and the F1-score of the simple LSTM was slightly better than bidirectional LSTM (91.61% vs. 90.4%).

### 4.2. User Authentication in Experiment 1 (a Targeted Box and Block)

In the case of user authentication in Experiment 1, as presented in Table 3, the best average F1-score and EER were reported in the LSTM models. However, considering that the false positive rate (FAR) results in more damaging authentication security issues than other metrics, such as false rejection rate (FRR), all ResNet models exhibited better performance in FAR than the LSTM models. In particular, ResNet-152 reported a minimum rate of FAR (1.67%). At the same time, its accuracy and F1-Score were equivalent to the LSTM models and greater than the rest of the ResNet models.

### 4.3. User Authentication in Experiment 2 (Smartphone Manipulation Scenario)

Table 4 summarises the average authentication performance results of Experiment 2. Unlike Experiment 1, the ResNet models outperformed the LSTM models for almost all metrics. Specifically, ResNet-152 achieved remarkable authentication performance, with an average F1 of 87.82% and an EER of 1.62%. Considering that the assigned object manipulation scenario in Experiment 2 is complex and realistic in comparison to Experiment 1, ResNet-152 better fits natural hand behaviour biometrics compared to the existing benchmark deep-learning models.

While the best average FAR was achieved using the bidirectional LSTM, with an average of 1.62%, its FRR value was considerably higher, with an average of 16.67%. This implies a high likelihood of rejecting legitimate users, which may cause serious usability concerns. A similar authentication tendency was observed in the simple LSTM and ResNet-101 models.

## 5. Discussion

We introduced a state-of-the-art deep residual network with a depth of up to 152 layers for user authentication in object manipulation and compared its performance against the benchmark LSTMs (simple LSTM and bidirectional LSTM). Overall, the experiments showed that both the ResNets and LSTM are acceptable for modelling user’s behavioural patterns for authentication tasks, with the best average accuracy of 96.31% and F1-score of 88.08%. Specifically, the ResNet models outperformed in age-group classification and user authentication in the category of complex object manipulation scenarios. In juxtaposition, the best performance of the LSTM models was observed in the user authentication for the simple box and block tasks.

The comparison confirmed that the ResNet model can be expected to deliver user authentication in a natural hand-object manipulation setting by capturing rich features for classifiers to improve their performance. We observed that more layers in residual networks tend to show better performance in user authentication, without showing degradation problems (i.e., increasing the depth of a network leads to poorer performance for both the test and training data). In the present study, the 152-layer ResNet performed best, with an average accuracy of 95.95%, F1-score of 87.82%, false acceptance rate (FAR) of 3.23%, and equal error rate (EER) of 1.62%.

We observed that ResNet-152 did not outperform the benchmark studies that employed deep learning architectures, notably, the LSTM model based on continuous user authentication using smartphone sensors [4] and three-layered CNN model for human activity identification [13], achieving an F1-score of 97.52% in [4] vs. 93.70% in [13] vs. 91.82% in the present study. Notwithstanding, we still expect that the proposed user authentication method utilizing wearable IMU systems and the deeper ResNet models has the potential to demonstrate robust authentication performance considering the challenges of the present study in terms of experimental protocol, sampling periods, and training data volume.

First, the finger-and wrist-mounted IMU-based system enabled the data collection of complex hand behaviour data containing rich and non-linear features. Secondly, unlike recurrent neural networks (e.g., LSTMs), CNN-based models have proven to be efficient in learning hierarchical hidden representations, with increasing levels of abstraction in their subsequent layers [21]. The state-of-the-art CNN-based ResNet model has a stronger modelling capacity, and the addition of more layers will aid in the progressive learning of more complex features, as compared to conventional CNN models [24]. Thirdly, concerning the sampling period of wearable sensor data, Zhu et al. [13] used a deep learning-based authentication model that required 8 s to build hand-object manipulation sequence data; the present study only required a 1.5 s window to build the hand sequence data. Our approach therefore allows a more instantaneous response within the authentication session and better interprets more complex and short-term hand-object manipulation, with a relatively lower computational load.

**Figure 7:**
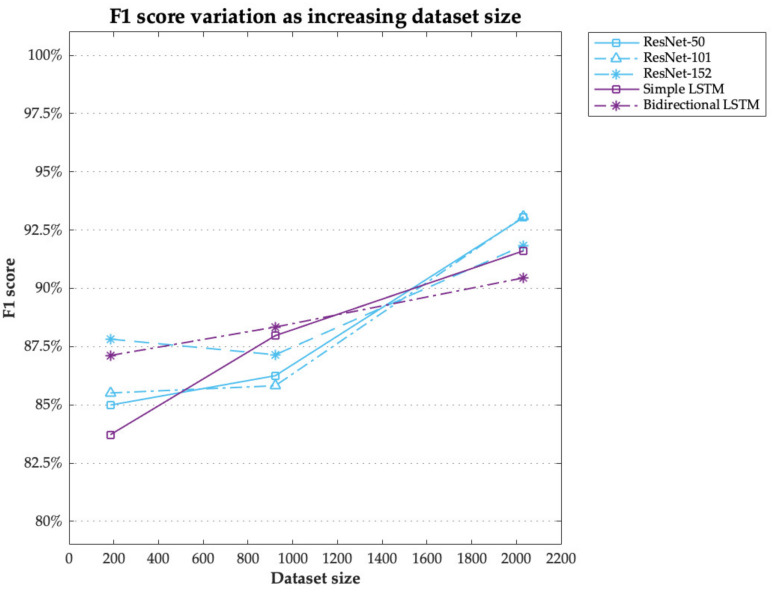
F1 score variation of deep learning models as dataset size increases.

Finally, considering that the performance of a deep-learning algorithm directly depends on the amount of training data, Figure 7 demonstrates how the performance of the present models improved with the increase in the size of the dataset. The marked data points in Figure 7 are based on the two experiments conducted in the present study: 2032 simple hand behaviour datasets for age-group classification in Experiment 1, 922 simple hand behaviour datasets for user authentication in the same experiment, and 185 complex hand behaviour datasets for user authentication in Experiment 2.

In both deep neural networks, the F1 score tended to increase with the volume of the training dataset. Although the graph conveyed mixed interpretation concerning the overall performance between the ResNets and LSTMs in the case of a small training dataset, there is a clear tendency of the ResNet models’ performance dramatically improving as the volume of the training dataset increases.

In comparison to the benchmark LSTM models, the ResNet models exhibit several advantages in learning complex hand behaviour with temporally rich features for continuous user authentication. First, considering that the ResNet model can better understand short-term sequences compared to sequential analysis-based models, such as LSTM models [31], its shorter sequence length for user authentication results in a less intrusive user experience for behaviour biometrics [4]. Secondly, to cope with the ever-increasing human behaviour data derived from smart devices, such as wearable devices, smartphones, and the Internet of Things (IoT), ResNet models can be a promising approach in terms of the algorithm’s efficiency and accuracy.

## 6. Conclusions and Future Works

### 6.1. Conclusions

Our research presents an implicit and continuous user authentication model via hand-object manipulation behaviours through the use of deep learning models. This study provides the following three contributions. Firstly, to the best of our knowledge, this is the first study to employ state-of-the-art deep residual network models (i.e., ResNets) for hand behavioural biometrics and verify their excellent authentication performance, compared to the benchmark deep learning model: long short-term memory models (simple LSTM and bidirectional LSTM). Secondly, we conducted a comprehensive investigation of hand-and finger-issued behaviour for authentication using a wearable IMU system and built a dataset using the data of 44 subjects, including high-fidelity sensory data from different age groups and simple–complex daily object manipulation scenarios. Secondly, using sensory data of 1.5 s, which is a relatively short sequential timeframe for behavioural biometrics, both ResNet and LSTM models demonstrated acceptable levels for user authentication, with an accuracy over 90% and F1-score of 85% for all scenarios. In particular, the experimental results showed the excellent performance of the ResNet models in the category of complex hand behaviour scenarios. The 152-layer ResNet performed best, with an average accuracy of 95.95%, F1-score of 87.82%, false acceptance rate (FAR) of 3.23%, and equal error rate (EER) of 1.62%.

The contributions of this paper are as follows:

- We proposed a user authentication model for object manipulation using a wearable motion sensor and deep learning models. A finger-and wrist-mounted IMU-based system collected complex hand behavioural data;
- We compared the state-of-the-art CNN-based deep residual network models (i.e., ResNets) and RNN-based long short-term memory networks (LSTMs) for user authentication via hand-object manipulations;
- We conducted comprehensive experiments in different age groups and simple and complex daily object manipulation scenarios to increase ecological validity. ResNet models outperformed user authentication in the category of complex hand behaviour scenarios compared to LSTMs. In particular, the 152-layer ResNet performed best, with an average F1 score between 87.15% and 91.84%, an equal error rate (EER) between 1.62% and 5.82%, and a false acceptance rate (FAR) between 1.67% and 3.23%.

### 6.2. Limitations and Future Works

There are two major limitations in this study that could be addressed in future research.

The first issue is how to enhance usability for authenticating users for real-time authentication. To address this issue, the model training time for real-time authentication needs to be reduced. We considered how to make the best use of the raw data to obtain hand dynamics features and the training of the features to build an authentication model for user identity verification. Some researchers have suggested several solutions to improve the model training time for real-time authentication, for instance, the minimisation of computational complexity [32] and the distributed computation sources [5]. Winoto et al. [32] proposed a near-real-time system by utilising depth-wise separable convolution in CNNs, which greatly reduced the parameter and computational complexity. Its performance is comparable with the ResNet models shown in the present study, while the computation time was decreased.

On the other hand, the present study did not consider allocating computation sources separately, which will theoretically make processing time faster. The present study employed a single computation source to train, verify, and authenticate user’s hand-object manipulations, resulting in a few minutes needed to train the proposed authentication models. Cloud server-based authentication model training and the model distribution to users could actually increase the efficiency on computation source utilisation and reduction in training time [5]. With a cloud server built in a high-performance computation environment, the proposed deep learning-based authentication model can further optimise its parameters continuously as new hand-object manipulation data are registered.

The second issue is how to improve the authentication performance. Figure 7 shows the tendency that the F1-scores in all deep-learning algorithms show better performance in relatively larger training datasets. Most importantly, the ascending tendency was relatively greater in the ResNet models compared to the LSTMs. Along with the increase in F1-score, FAR and EER of the deep learning-based models tended to decrease because these metrics are closely associated with F1-score (See Figure 6). Consequently, the proposed authentication system urgently needs to develop a set of design techniques enabling a large collection of hand-object manipulation data to train. One of the suggested solutions is to build a cloud server that automatically collects hand-object manipulation data. In so doing, the training dataset can be collected at a large scale for real-time authentications. Similarly, Abuhamad et al. [4] demonstrated that a deep learning-based authentication model could be progressively optimised when a sufficient amount of human behaviour data are used during the model training session. For instance, with five-day smartphone usage data from 84 subjects, they achieved an EER of less than 1%.

Furthermore, because wearable devices and IoTs are widely used in our daily lives, it is possible to capture users’ hand behaviours and enable silent user authentication, based on raw data collected from diverse forms of motion sensors that can be attached to either parts of the human body or objects. Given that humans can demonstrate dozens of hand movements, with different traits for each different object with which they interact [33], a major challenge in future work will lie in the testing of a methodology within an IoT environment where a system continuously monitors or authenticates users as part of daily activity and improves the accuracy and user-friendliness of the behaviour biometrics method.

## Figures and Tables

**Table 1:** Performance metrics.

Metric	Equation
Accuracy	$\left(TP+TN\right)÷\left(TP+FN+FP+TN\right)$
Recall	$\left(TP\right)÷\left(TP+FN\right)$
Precision	$\left(TP\right)÷\left(TP+FP\right)$
F1 Score	$2\times \left(Recall\times Precision\right)÷\left(Recall+Precision\right)$
False Acceptance Rate (FAR)	$\left(FP\right)÷\left(FP+TN\right)$
False Rejection Rate (FRR)	$\left(FN\right)÷\left(TP+FN\right)$
Equal Error Rate (EER)	$\left(FAR+FRR\right)÷2,where\;\left|FAR-FRR\right|\;is\;minimum$

TP (true positive), TN (true negative), FP (false positive), FN (false negative).

**Table 2:** Performance of age group classification in the targeted box and block test (Experiment 1).

Model	Accuracy	Recall	Precision	F1 Score
ResNet-50	92.96	93.12	92.96	93.04
ResNet-101	93.05	93.11	93.05	93.08
ResNet-152	91.82	91.86	91.82	91.84
Simple LSTM	91.58	91.63	91.58	91.61
Bidirectional LSTM	90.44	90.46	90.44	90.45

**Table 3:** Performance of user authentication in the targeted box and block test (Experiment 1).

Model	Accuracy	Recall	Precision	F1 Score	FAR	FRR	EER
ResNet-50	95.87	85.00	87.69	86.25	2.18	15.00	6.64
ResNet-101	95.76	84.28	87.54	85.82	2.18	15.72	6.32
ResNet-152	96.20	84.28	90.89	87.15	1.67	15.72	5.82
Simple LSTM	96.20	91.43	84.95	87.97	2.95	8.57	3.71
Bidirectional LSTM	96.41	89.29	87.52	88.34	2.31	10.71	4.85

**Table 4:** Performance of user authentication in the smartphone manipulation scenario (Experiment 2).

Model	Accuracy	Recall	Precision	F1 Score	FAR	FRR	EER
ResNet-50	94.59	91.67	81.25	84.99	4.84	8.34	3.23
ResNet-101	95.27	83.33	88.69	85.51	2.42	16.67	11.56
ResNet-152	95.95	91.67	84.52	87.82	3.23	8.34	1.62
Simple LSTM	94.59	83.33	84.28	83.56	3.23	16.67	11.56
Bidirectional LSTM	95.94	83.33	91.67	87.12	1.62	16.67	9.95

## Data Availability

Data sharing is not applicable to this article.
